# Sugar Content in *Arbutus unedo* L. Fruit and Its Relationship with Climatic and Edaphic Characteristics

**DOI:** 10.3390/plants13233383

**Published:** 2024-12-01

**Authors:** Luciano Chá Chá, Sandrine Ressurreição, Libânia Oliveira, Sandra Santos, Manuel Nunes, Maria Vidal, Jorge Varejão, Filomena Gomes

**Affiliations:** 1 Polytechnic University of Coimbra, Rua da Misericórdia, Lagar dos Cortiços, S. Martinho do Bispo, 3045-093 Coimbra, Portugal; llucianojcc@gmail.com (L.C.C.); sandrine@esac.pt (S.R.); libaniasofia93@gmail.com (L.O.); sds@esac.pt (S.S.); mnunes@esac.pt (M.N.); jvarejao@esac.pt (J.V.); 2 Research Center for Natural Resources, Environment and Society (CERNAS), Polytechnic University of Coimbra, Bencanta, 3045-601 Coimbra, Portugal

**Keywords:** annual precipitation, annual temperature, fructose, limestone-derived soil, orchard establishment, photosynthesis, schist-derived soil, strawberry tree, Thornthwaite Method, xerothermic index

## Abstract

This research was carried out as part of a program for the conservation and improvement of the strawberry tree. Accessions’ prospecting was conducted in different Portuguese provenances. Accessions (204) were identified, and mature fruits were collected in autumn. The sugar contents in the fruit pulp (glucose, fructose, maltose, sucrose) were analyzed by HPLC and the correlation between the average sugar content, climatic classification (Thornthwaite Method and Xerothermic Index), and edaphic characteristics was investigated. The predominant sugar was fructose, which ranged from 7.89 ± 0.55% to 17.01 ± 1.46% (f.w.), respectively, under an Attenuated Thermomediterranean climate at limestone-derived soil and an Accentuated Mesomediterranean climate at schist-derived soil. The lowest sugar contents, linked to a reduction in photosynthesis, were found: (1) in the north, despite soil water availability, due to the low temperature, high precipitation and short photoperiod, particularly during the fruit maturation (autumn); (2) in the south, due to the excessive number of dry days, linked to low soil water availability during the active growth period, particularly at limestone zones prone to water retention. The relationship found between the total sugar content and climate classification by the Xerothermic Index allows to enhance fruit production, advise on the establishment of new orchards and restore natural areas.

## 1. Introduction

The use and appreciation of native edible plants, both wild and cultivated, have been essential to the development of numerous cultures worldwide, particularly in the Mediterranean region, where these plants play a vital role in the health and well-being of local populations [1,2,3,4]. *Arbutus unedo* L., commonly known as the strawberry tree, is a small tree or shrub of the Ericaceae family, typical of Mediterranean climates [5]. Highly resistant to harsh conditions, it grows spontaneously in various regions of Portugal, predominantly south of the Tagus River, particularly in mountainous areas, such as the *Caldeirão* and *Monchique* [6]. This plant is of great ecological interest due to its numerous environmental benefits [7]. The successive flowerings during winter and its evergreen canopy contribute to biodiversity, serving as food and shelter for bees, birds, and small mammals, while its branched root forms mycorrhizal associations with a large group of fungi [8]. This symbiotic community plays an essential role in soil stabilization and erosion control. It serves as a carbon and water reservoir and contributes to the formation of intraspecific associations, as evidenced by the example provided by Richard et al. [9] with *Quercus ilex*. In addition, strawberry tree ability to regenerate after fire, makes it an ideal candidate for reforestation projects to promote plants survival in Mediterranean ecosystems [10], in the restoring of degraded soils [11], and used as a broadleaved green firebreak around villages and infrastructures, particularly in the wildland-urban interface [12].

From an economic perspective, the strawberry tree is one of the most important and widely recognized shrubs in the Mediterranean basin. It is valued for its many applications, including an ornamental plant, a melliferous plant [13], for its fruits [14] and wood and for medicinal purposes [3,4,15,16]. This multifunctional species has a wide range of applications in various industries, including nutraceuticals, pharmaceuticals, cosmetics [3,17,18,19], and food [20], due to its antiseptic, diuretic, and antioxidant properties [1,21,22]. The fruit of the strawberry tree, an edible berry, contains a high proportion of sugars, with the following fructose content (dominant sugar), expressed in fresh weight 3.65–12.34%, 16.62 ± 0.50%, 5.52–8.44% (*w*/*w*, f.w.) [23,24,25], and reducing sugars of 16.8–18.6% (*w*/*w*, f.w.) [26]; and, expressed in dry weight, have the following fructose contents: 27.8 ± 0.32, 24.09 ± 5.37%, 20.08 ± 0.2%, 8.73–13.08% (*w*/*w*, d.w.) [27,28,29,30], and reducing sugars: 6.98–9.27% (d.w.) [31]. Other nutrients were found, including minerals [32], polyphenols [1,3,23], organic acids, volatile compounds [33], and antioxidant compounds [4] such as vitamins [34,35]. The composition of these compounds varies with the ripening stage of the fruit, as has been shown in numerous studies [14,18,19,22,33,35,36]. However, according to Morales [37], despite numerous studies and references to the use of the *A. unedo* in traditional folk medicine, further studies are needed to evaluate biological activity, animal studies and clinical trials. Similarly, apart from the well-established distillery industry, there are few advanced technologies for other uses of fruit, sclerenchyma and leaves, as well as efficient technology for extraction, purification and isolation of bioactive compounds, followed by appropriate scientific validation for subsequent use and dissemination.

Fruits can be consumed fresh, made into jams, jellies, and preserves, or fermented and distilled to produce alcoholic beverages, such as strawberry tree brandy [38,39]. This distinctive and aromatic spirit beverage is produced through the distillation of fermented fruits, a practice that is particularly prevalent in the southern and central regions of the country, representing a sustainable agroecological practice [40]. Despite the production costs of “*Aguardente de medronho*”, as it is known in Portugal, the market value of this product ensures its profitability, thereby enhancing the social and economic significance of the strawberry tree. Currently, three denominations of origin are recognized: *Medronho do Algarve*, *Medronho do Buçaco* and *Medronho do Sudoeste*.

The fruits covered with conical papillae (sclerenchyma tissue) are orange to red, when mature, and ready to be harvested, during autumn. It takes one year from flowering to fruit ripening, which is a long period, with the fruits under environmental stress (cold, frost, wind and then heat and water stress). During autumn, the plant displays both different physiological stages, ripening fruits and flowers [41].

In a three-year study of fruit harvesting in two natural forest areas in Spain, with differing environmental conditions [23] revealed significant variability in sugar content, with sucrose, glucose and fructose exhibiting an increase order of concentration. Similar results in physicochemical composition and the content of specialized metabolites were reported for fruits harvested in different ecological conditions in Croatia [42]. Thus, and considering the increased interest in this crop in Portugal, and the potential uses of its fruits, it is relevant to better understand the relationship between sugar content in *A. unedo* fruits and climatic and edaphic characteristics. The knowledge and understanding of the dynamics in the fruit sugar content-soil-climate system will allow us to advise either plant field establishment (e.g., sun exposure) or fertilization program [43], with the objective of promoting fruit production and quality. For this purpose, a total of 204 accessions were identified through continental territory from North to South and mature fruits were collected during the autumn season. Fruits were processed to obtain fruit pulp free of seeds and sclerenchyma tissue. Then, sugar contents were analyzed using high-performance liquid chromatography (HPLC), with the objective of discerning the percentage composition of glucose, fructose, maltose, and sucrose. Furthermore, in order to evaluate the relationship between the sugar levels observed in the fruits and the edaphoclimatic characteristics of the accession site, the correlation between the average sugar levels, the climatic classification (determined by the Thornthwaite Method and the Xerothermic Index) and the edaphic characteristics, mainly the soil and its mother-rock, was studied.

As potential limitations of this study, we can mention that it will be relevant to show the relationship between sugar content and other interesting agronomic parameters such as fruit size, total soluble solids (°Brix), fruit firmness, excessive presence of sclerenchyma (inconvenient for the fresh fruit market) and finally its relationship with genetic diversity analysis. Work is already underway on some of these topics.

## 2. Results

### 2.1. Analysis of Fruit Sugar Content of Accessions from Different Provenances

Table 1 presents the sugar content characterization of the fresh pulp of the *A. unedo* fruit, from the 204 accessions of different provenances. The concentrations of sugars (expressed in grams per 100 g of fresh pulp) decreased in the following order: fructose, the most abundant sugar, followed by glucose and residual contents of maltose and sucrose. The mean values of fructose ranged from 7.89 ± 0.55% to 17.01 ± 1.46%, followed by glucose, which ranged from 3.98 ± 0.11% to 6.80 ± 0.26%. Then, residual values of maltose and sucrose were observed, ranging from undetected to 3.73 ± 0.53% and from undetected to 1.58 ± 0.15%, respectively.

Regarding the mean value of sugar per provenance, the location *ODM-Cortes Pereira* exhibited the highest mean values for fructose (17.01 ± 1.46%), reducing sugars (26.56 ± 2.31%) and total sugar (27.07 ± 2.30%). However, no statistically significant differences (*p* > 0.05) were observed regarding the remaining provenances that received producers’ support for the identification of accessions, with the exception for *ODM-Trancão* and *ODM-Cerca dos Pomares* and *Penha Garcia* (Table 1). In contrast, the lowest concentration of the reducing sugars, mainly fructose and glucose, was observed in the *TM-Fisgas de Ermelo*, *TM-Mosteiró* and *Barrocal*. The former two cases are associated with the high precipitation rates and the lower average temperatures, while the latter exhibits the highest xerothermic index/number of dry days (Table 2).

### 2.2. Sugar Content and Its Relation to Climatic Classifications and Edaphic Characteristics

The correlation between the sugar content of fresh pulp of the *A. unedo* fruit and the climatic classification of the respective accession regions, as determined by the xerothermic index, is presented in Table 3.

In accordance with the 2.1 preceding discussion, Table 3 illustrates that the highest mean values of reducing and total sugar (respectively, 19.61 ± 0.46% and 20.39 ± 0.47%) and fructose (12.79 ± 0.29%) were observed in the provenance group classified as *Accentuated Mesomediterranean* (133 accessions), which includes the *ODM-Cortes Pereira* provenance and its 15 accessions. Furthermore, these elevated sugar contents are statistically significantly different (*p* < 0.05) when compared to the provenances from other climatic classifications (71 accessions).

With regard to the glucose, the highest mean value (5.40 ± 0.12%) is also observed in the *Accentuated Mesomediterranean* region, despite no significant differences (*p* > 0.05) being identified when provenances from *Attenuated Mesomediterranean* (*TM-Mosteiró* and *S. Mamede*) were compared (Table 2 and Table 3).

In contrast, the lowest fructose value was observed in *Barrocal*, a provenance classified as *Attenuated Thermomediterranean* (7.89 ± 0.55%), with no significant differences (*p* > 0.05) identified when compared to the *TM-Fisgas de Ermelo* provenance (*TM-FE*) from a *Sub-Mediterranean* climate (8.66 ± 0.42%) (Table 3). The climatic classification based on the xerothermic index reveals that *Barrocal*, the southernmost provenance under consideration in this study, is not included in the climatic region of the remaining southern provenances. This exclusion may be attributed to the highest number of dry days (118.18) observed in this region. However, the second lowest fructose value was observed in the *TM-FE*, situated at the northernmost point of Portugal and in a *Sub-Mediterranean* climate. This was despite the lowest number of dry days (42.40), but due to the highest annual precipitation value (1390.5 mm vs. to *Barrocal*, 697.0 mm) and the lowest annual temperature (13.4 ºC vs. 15.9 ºC).

Table 4 presents the classification of the various provenances of the *A. unedo* accessions, differentiated into 6 climatic regions according to the Thornthwaite climatic classification [49], and shows the correlation between the sugar content of the fresh pulp of the fruits and the corresponding climate classification of the regions of origin.

According to this new classification criterion, the highest mean values were observed in C1 B′2 s2 a′ climatic region corresponding to the 15 accessions of *ODM-Cortes Pereira* provenance (Table 1 and Table 4) with fructose 17.01 ± 1.46%, reducing sugars of 26.56 ± 2.31% and total sugar of 27.07 ± 2.30%, with significant differences (*p* < 0.05) compared to the values of reducing and total sugars observed in all the other provenances (Table 4). Although, for fructose values no significant differences were found when compared to the provenances of *ODM-Cerca dos Pomares* and *Cadaval* (7 accessions, Table 2 and Table 4), both included in C2 B′2 s a′, according to the Thornthwaite method (Table 2 and Table 4). Thus, in the Thornthwaite classification the *ODM-Cortes Pereira* provenance is distinguished from the other southern provenances. On the contrary, *Barrocal* was included together with *ODM-Pomba* and *ODM-Nave Redondo*, in the same climatic region C2 B′2 s2 a′ (46 accessions in total, Table 2 and Table 4).

Also, for the lowest fructose results, the Thornthwaite classification does not discriminate Barrocal provenance, for which the lowest reducing and total sugar contents were assigned to the strawberry tree fruits in the climatic regions B4 B′2 s a′ and B3 B′2 s2 a2, corresponding to the provenances of *TM-FE* and *TM-Mosteiró*, respectively (Table 4). Both provenances show humid climates with a high water index (B4, B3), with moderate (s) to large (s2) water deficit in summer, respectively *TM-FE* and *TM-Mosteiró*.

As presented in Table 5, the results of the analysis indicate that precipitation, xerothermic index, soil pH, climatic classification according to xerothermic index and humid index (Ihu) (*p* < 0.05) are the independent variables that most influence on total sugar content. A statistically significant decrease in total sugar content is observed with the concomitant increase of these variables (*p* < 0.05), apart from the Ihu. Furthermore, it can be observed that annual precipitation and Ihu are the most significant independent variables, as both have the highest absolute values for the coefficient of determination (b). Thus, the results of the linear regression analysis corroborate the findings presented in the preceding discussion. According to the climatic classification based on the xerothermic index, the lowest total sugar values were observed in the two northernmost and in the southernmost origins included in this study. It was observed that the highest annual precipitation, associated to the lowest number of dry days (lowest xerothermic index), was recorded in the provenances *TM-FE* and *TM-Mosteiró*. Conversely, in *Barrocal*, despite the highest number of dry days, as indicated by the highest value of the xerothermic index, the lowest Ihu value and low precipitation resulted in the lowest total sugar content. This was a consequence of the limited availability of water, which in turn impeded photosynthesis and the synthesis of sugars.

**Figure 1:**
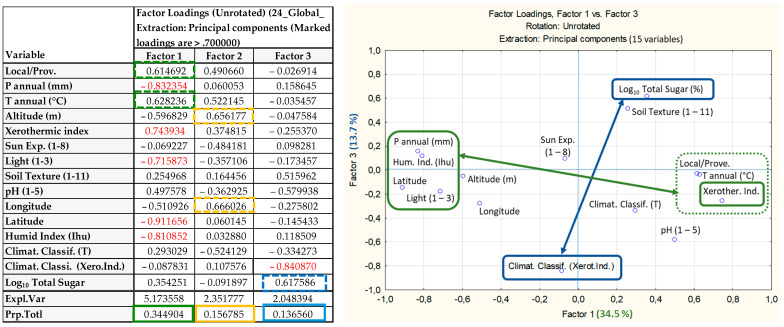
Interaction among the total sugar content (% f.w.), site features (latitude, longitude, altitude), edaphoclimatic characteristics and climatic classifications (Thornthwaite and xerothermic index) by PCA analysis (see left table). Note: The variables are associated with each factor 1–3, according to the coefficients’ factor loadings, presented on the left table (green, yellow and blue for factor 1, 2 & 3, respectively). Moreover, the highest factor loadings (>0.70) are marked in red (on table) as they are relevant to explain the total variance expressed by each factor (1 to 3), as well as correlated among them (direct or inversely/on opposite sides). The figure shows the interaction between factor 1 and factor 3, in green and blue, respectively, as well as the line colors, that show the relationship between the variables associated with the same factor, directly or inversely.

A negative coefficient, meaning an inverse relationship, was identified between the soil pH parameter (1–5, corresponding to pH < 4 to pH > 9) and the dependent variable. In the present study, only one provenance had a soil pH value of 4, indicating a soil pH between 7.5 and 9.0, which was *Barrocal*. Notwithstanding the lowest sugar content observed in the Barrocal (20 accessions; Table 1), three out of four provenances with a soil pH value of 2 (pH = 4–5.4; *TM-FE*, *S. Mamede* and *Penamacor*) exhibited total sugar contents that were also inferior to the general mean value (18.47 ± 0.44% f.w.). The exception is Monchique (21.34 ± 0.97% f.w.), confirming the relevance of producers to identify the plants. To analyze the relationship among the total sugar content (Log_10_% f.w.), site features (provenance, latitude, longitude, altitude, sun exposure, light conditions), edaphoclimatic characteristics (annual precipitation and temperature, humidity index, xerothermic index, soil texture, pH) and both climatic classifications (Thornthwaite method and xerothermic index) a principal component analysis (PCA) was performed, considering a total of 15 variables (Figure 1).

The objective of PCA is to establish a relationship between the 15 variables under investigation. Factors 1 to 3 (present in the table on the left in Figure 1) demonstrate the correlation between the variables and quantify the variance explained by each factor (Factors 1 to 3– % total variance, last row in the table). To facilitate interpretation, each factor (1 to 3) can be plotted (e.g., factor 1 on the x-axis and factor 2 on the y-axis). Factor 1 corresponds to a multiple regression of the 15 variables (y = a + b_1_ x_1_ + … + b_15_ x_15_), as shown in the table, together with their respective coefficients (b), named factor loadings. Thus, high coefficients (factor loadings > 0.70, shown in red) indicate which variables are strongly correlated, directly (if with the same sign) or inversely (if with opposite signs), and also associated with the variance explained by the respective factor (e.g., Factor 1-34.49%). The factors (1 to 3 …) indicate, in descending order, their importance in explaining the variance observed for the set of variables analyzed. Principal component analysis (PCA) accounts for 63.82% of the total variance observed in the 15 variables. Of this, 34.5%, 15.7%, and 13.7% are associated with the variables related to factors 1, 2, and 3, respectively (see Figure 1, left table).

Factor 1 shows a direct relationship between annual precipitation (mm), light conditions (levels 1–3), latitude, and humidity index (Ihu). All these variables show a high negative coefficient factor loading (>−0.70) and are inversely related to the xerothermic index (>0.70) and to annual temperature and provenance (factor > 0.61).

Factor 1 also shows that the plants prospected and harvested in light condition 3 (under the canopy of forest trees) are mainly located in the northern regions of the Tagus River (highest latitude). In this area of the country, the main land use is forest to produce *Eucalyptus globulus* and *Pinus pinaster* wood, which is one of the most important economic activities, while the strawberry tree is a wild plant located in the lower stratum [50,51]. However, either small forest producers (mainly own land) or cellulose companies, are becoming aware of the importance of autochthonous species for the protection of forests and interurban spaces [52]. In Portugal, *Arbutus unedo* is becoming one of the most autochthonous species by stakeholders in nurseries to be established as orchards and as green firebreaks [12].

Factor 2 shows the direct relationship between longitude and altitude, showing that plants identified and harvested in inland (higher longitude) are predominantly located in mountainous regions (higher altitude).

Factor 3 confirms the relevance of climate classification by the xerothermic index (high factor loading) in total sugar content (>0.61; left Table on the Figure 1), indicating that climate classification by the xerothermic index, compared to Thornthwaite Climatic Classification, allowed a better discrimination of the potential of the different provenances on fruit sugar content (considering a total of 14 provenances and 204 accessions). These outcomes corroborate the results presented in Table 1, Table 3, Table 4 and Table 5.

Overall, both analyses (multiple regression and PCA) confirm the previous observations. The lowest sugar contents were observed in the two northernmost origins (*TM-FE, TM-M*) and in the southernmost origin (*Barrocal*). In the northernmost region, high precipitation, lower temperature and shorter photoperiod are limiting factors, especially during the fruit maturation period. In the southernmost region, the greater number of dry days and the soil pH, associated with the mother rock (limestone) and clay loam, are the main limiting factors, that reduce water bioavailability, especially during active fruit growth. Moreover, both analyses confirm the inverse relationship between sugar content and climatic classification by xerothermic index, proving that this is an effective tool for distinguishing the potential of each provenance for fruit sugar production, as well as for identifying regional limitations, providing guidance for the establishment of new orchards and facilitating the restoration of natural areas.

## 3. Materials and Methods

### 3.1. Characterization of Accession Prospecting Sites/Provenances

This research was carried out as part of the program for the conservation and genetic improvement of the strawberry tree, with the aim of prospecting and collecting the genetic diversity [53] of cultivated and wild plants for their inscription in the Portuguese Plant Germplasm Bank (https://www.iniav.pt/bpgv, accessed on 30 September 2024), and in the GrinGlobal platform (https://www.grin-global.org/, accessed on 30 September 2024). A total of 204 accessions were prospected and harvested in 14 sites/provenances, distributed from the north to the south of the country and from the inland to the coast.

**Figure 2:**
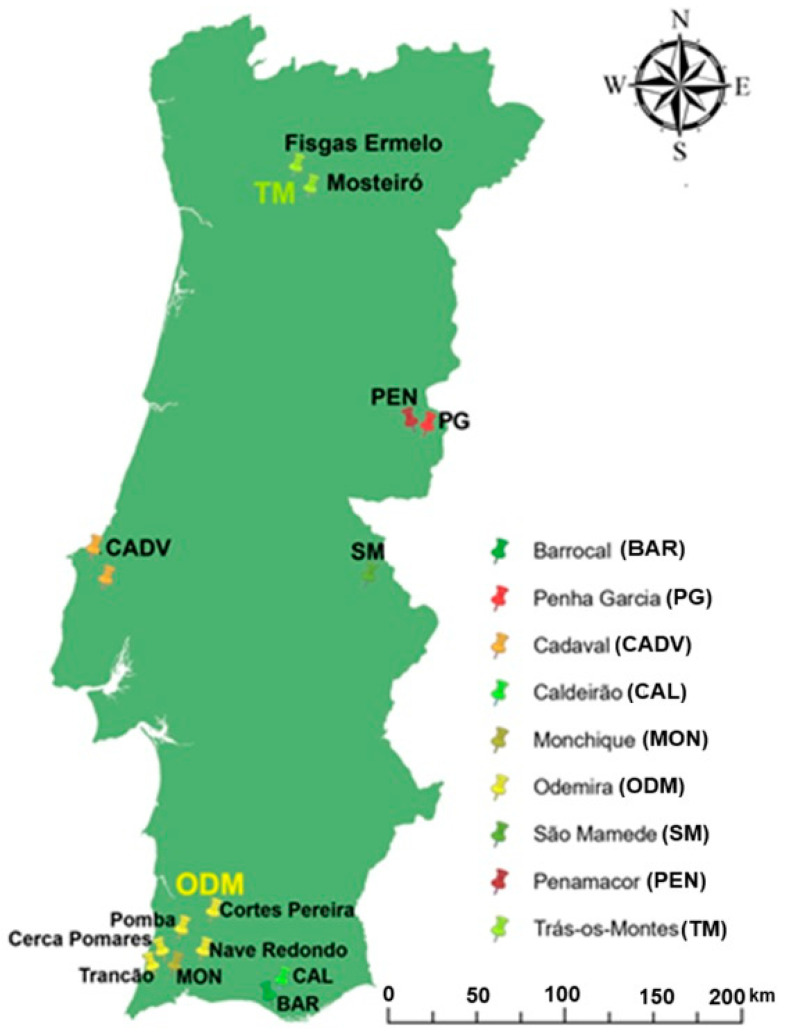
Geographical distribution of A. unedo accessions harvested in the study area in Portugal.

The identification of prospective sites was based on the application of two distinct criteria. The first criterion involved the inclusion of provenances that have a historical and economic tradition of harvesting fruit for brandy production [54]. The second criterion involved provenances representative of the diversity of the species excluding sites that had already been characterized in previous studies [55,56,57,58]. In this way, accessions of cultivated and wild plants (in non-irrigated orchards and natural areas, respectively) were identified by producers for their fruit production and quality, as well as accessions from wild areas where there is no tradition of economic exploitation of the species, but which are of potential interest to small farmers, particularly in mountainous regions, characterized by a higher risk of desertification and forest fires. In these areas, the species can play an important role, providing a new source of income and contributing to population settlement, while increasing biodiversity and conserving the soil. Figure 2 shows the distribution of the different accessions according to the 14 provenances (Table 2).

Each accession was characterized (Table 2) by geographical coordinates, altitude, sun exposure (1–8), light conditions (1–3), lithology [44], soil [46], pH (1–5), soil texture (1–11), location (inland vs. coast), mean annual precipitation and temperature (mm and °C; from the climatological normal corresponding to the period 1951–1980 [47], humidity index (Ihu), xerothermic index [48] and Thornthwaite climatic classification [49].

The sun exposure scale, which ranges from 1 to 8, represents in increasing order from (1) North-Northeast, (2) Northeast-East, (3) East-Northwest, (4) Northwest-North, (5) South-Southeast, (6) Southeast-West, (7) West-Southwest, (8) Southwest-South, and 4.5 to hilltop (no sun exposure and windy). The sun exposure is a relevant factor as it has been demonstrated to interfere with entomophilous pollination of flowers and the maturation of fruits, both processes occurring during the autumnal period in successive years [41]. The S-SW exposures have been shown to be conducive to flower pollination, a reduction in fruit drop during the winter period, and an increase in the number of fruits per cluster when compared to the N-NE-E-NW exposures [59]. Light conditions range from (1) direct sunlight, (2) indirect sunlight to (3) understorey. In addition, the oceanic influence was also considered in the variable location, with sites situated less than 16 km (in a straight line) being categorized as coastal. Soil pH is reported in an ascending order (1–5), from very acidic soils with a pH below 4 to alkaline soils with a pH above 9; and finally, soil texture is presented 1 to 11, according to the guidelines of the collection form for plant material from wild plants from the Portuguese Plant Germplasm Bank (1-sandy; 2-loam; 3-sandy loam; 4-clay loam; 5-clay; 6-silt; 7-silt-clay; 8-sandy loam; 9-loamy; 10-sandy clay; 11-highly organic).

The Thornthwaite Climate Classification characterize climate developing soil water balance accounting soil storage inflows (precipitation) and outflows (evapotranspiration and water excess) [60]. Evapotranspiration component is achieved by the calculation of the potential evapotranspiration (PET), that depends on the monthly temperature and an adjustment factor given by the latitude [60]. The effective soil outflow is the real evapotranspiration (RET), obtained according to the soil water potential differential (PET–P), leading to months in which the potential value is reached (wet season) and months in which the actual value is lower than the potential (dry season). In Thornthwaite classification it is usual to consider a total soil water storage capacity of 100 mm, which was used.

The evolution of soil water storage allows the identification of periods of excess and deficit of water for each month and their quantification. These values are used to calculate several water indexes, as a resume of the balance. Hydric Index (IH) is the most important, which represents the global behavior of the climate in hydrological terms.

This is represented by the 1st letter of the Thornthwaite climatic classification (ranging from A to E, corresponding from *Perhumid* to *Arid*) [49]. According to this, the provenances of this study, situated in the northern and central regions of Portugal, show *Humid* and *Rainy Sub-Humid* climates (B and C2) characterized by moderate to severe water scarcity during the summer months. On the other hand, the southern region is characterized by *Dry Sub-Humid Climates* (C1), showing moderate to high water surplus during the winter season. Also, in both regions, the humidity increases in proximity to mountain ranges and decreases with distance from the Atlantic coast. The 2nd letter of the Thornthwaite climatic classification refers to thermal efficiency, which is the sum of *potential evapotranspiration (PET)* ranging from A′/*Megathermal* to E′/*Frost* (corresponding to ∑PET ≥ 1140 mm vs. <142 mm, respectively). According to this, all provenances in this study are classified as B′2 (*Mesothermal*), corresponding to the range 712 ≤ ∑PET < 855 mm. The 3rd letter of the Thornthwaite climatic classification refers to the Aridity index and Humidity index for *humid* (A, B, C2) and *dry* (C1, D, E), climates respectively. Accordingly, in Portugal, mainly in the northern and mountainous areas: (i) *Humid* and *Subhumid* climate group (1st Letter A, B or C2): s means *moderate water deficiency in summer*; and s2 corresponds to *severe scarcity of water in summer*); (ii) Dry climate group (1st letter C1 or D): s means *moderate excess water in winter*; and s2 corresponds to *high water excesses in the winter* season. The 4th letter of the Thornthwaite climatic classification refers to the *summer concentration of thermal efficiency*, which is calculated in percentage of the sum of the potential evapotranspiration during the three months showing the highest temperature. According to this and due to Atlantic influence, the *summer concentration of thermal efficiency is low* (25–48%) for all provenances [49,60]. It should be noted, however, that extreme temperatures associated with heatwaves may occur during the summer months, especially in the southern inland regions.

The humidity index (Ihu), derived from the Thornthwaite hydrological balance, is calculated using the formula:

Ihu = (Σ S/Σ PET) × 100,(1)

where “S” is the soil water excess

This index enables the characterization of the rainy season, which is of particular relevance for the study of dry climates, such as those prevalent in the southern regions (Table 2), where surplus water can be retained in the soil for utilization during subsequent dry periods [61]. In contrast, in humid climates (A, B and C2), which are characterized by a heavier rainy season, the humidity index is superfluous. Such conditions are observed in the northern area of the Tagus River (particularly in Minho and Douro regions) and in the mountainous regions in the south (Table 2).

The xerothermic index is a statistical measure, simpler to use and designed to indicate the climate level of dryness, that quantifies the number of days in each month when precipitation is less than twice the average temperature, as illustrated in the ombrothermic diagram. The number of dry days is calculated by summing the monthly xerothermic indexes, which represent the number of days when the plant is experiencing hydric stress, from a biological perspective [62].

The Xerothermic Index is useful for identifying dryness and assessing water availability for vegetation, while the Thornthwaite classification offers a more complex perspective of the climate and how evapotranspiration and precipitation determine soil moisture evolution in a region throughout the year. However, both classifications complement each other, providing a good characterization of the variability present on the Mediterranean climate in Portuguese area.

### 3.2. Methodology for Sugar Contents Analysis

The strawberry tree fruits were harvested from the different accessions during the autumn, at the phenological development stage L (*ripening*/*harvesting*), as previously proposed [59,63], based on the findings of studies conducted on other fruit species [64,65]. The fruits were placed in plastic boxes and transported under refrigerated conditions (4 ºC).

In the laboratory, fifteen fruits were randomly selected from each accession. The fruit pulp was extracted using a procedure previously proposed by J. Anastácio [66]. Briefly, a raw cloth was used to separate the seeds and sclerenchyma from the pulp, with the aim of obtaining a final weight of 200 g of fruit pulp for the analysis of sugar content by high-performance liquid chromatography (HPLC). For sugar content analysis, 2.5 g of the strawberry tree fruit pulp was dissolved in distilled water and stirred for 30 min. The mixture was then filtered through filter paper into a volumetric flask, and the final volume was adjusted to 100 mL. The resulting solution was further filtered through a 0.45 µm membrane filter before being injected into the HPLC system.

Sugar quantification was performed using high-performance liquid chromatography with refractive index detection (HPLC-RI). The system included an LC-1110 pump (GBC, Haymarket, NSW, Australia), LC-100 oven (Perkin-Elmer, Waltham, MA, USA), 830-RI refractive index detector (Jasco, Hachioji, Japan), and an HC-75 Ca++ 305 × 7.8 mm column (Hamilton, Reno, NV, USA). The mobile phase was ultrapure water with traces of sodium azide, at a flow rate of 0.6 mL/min and a temperature of 80 ºC. The quantification of glucose, fructose, maltose, and sucrose was carried out using BioUltra standards (Sigma-Aldrich, St. Louis, MO, USA). Data were collected using an Interface Hercule Lite (JMBS, Neuhaus, Germany) and processed with Borwin Chromatography Software, version 1.5, build 16 (Jasco-Borwin, Hachioji, Tokyo, Japan). Three replicates were performed per sample.

### 3.3. Data Processing and Statistical Analysis

The statistical analysis was conducted using *STATISTICA 12.0*. As there was no normal distribution of the sugar contents, these were transformed into Logaritms on base 10, after which the normal distribution was confirmed (*p* > 0.05, Shapiro-Wilks Test), followed by the analysis of variance (ANOVA) to evaluate the effect of different edaphoclimatic variables on the fruit sugar content. When significant differences were observed, the means were compared using the Tukey test (*p* < 0.05) [67]. Other complementary approaches were tested: multiple linear regression and a principal component analysis (PCA). For the multiple linear regression analysis, the effect of the different variables on the total sugar content (Log_10_) was evaluated, considering the edaphoclimatic characteristics as independent variables (Xi). To identify the linkage between the total sugar content and site characteristics a principal component analysis (PCA) was performed. For the PCA, 14 variables were analyzed to achieve a better understanding of the interactions between them and their significance level for the total variance.

## 4. Discussion

There have been several recent studies on the sugar content of *Arbutus* fruits harvested throughout the circum-Mediterranean region, as this is one of the main quality differentiating parameters of the fruit [1]. In our study, all samples (204) analyzed were found in decreasing order (*w*/*w* f.w.) of fructose, ranging from 7.89–17.01%, followed by glucose 3.98–6.80%, then maltose n.d. −3.73%, and sucrose n.d. −1.58%. Similar results, concerning the sugar content order, were found in Spain [23] with the following values (*w*/*w* f.w.), fructose (3.65–12.34%), glucose (2.34–6.50%) and sucrose (n.d. −0.48%). The same trend was observed in Turkey [27], where the average of fructose, glucose, sucrose and maltose, from young trees, were 27.8, 21.5, 1.8 and 1.11% *w*/*w* d.w., respectively. Vidrih et al. [24] also observed comparable sugar content when compared to us. Their findings were obtained from three wild-growing tree in Croatia and indicated a fructose concentration of 17.2% and glucose 6.2% (*w*/*w* f.w.). Our study finds similar results when compared to Alarcão et al. [29], considering the red mature fruits, which show higher content of fructose followed by glucose. On the contrary, Ait Lhaj et al. [30] found higher glucose contents in 2–7 trees of 12 provenances from five Moroccan regions (45 samples) in all provenances. They report glucose contents ranging from 11.57–15.17%, followed by fructose 8.73–13.08%, and with lower sucrose contents 4.16–8.11% (*w*/*w* d.w.). To the best of our knowledge, this was the only study to contradict the order of sugar content, reporting higher glucose content than fructose content.

A potential confounding factor for the results observed is linked to the support of producers during accessions identification, that is relevant for finding the best plants (with higher sugar content), which happened in the southern accessions, where the exploitation tradition is older than in the north [1]. So, three out of the fourteen provenances of our study (37 out of 204 accessions) showed an average fructose content above the upper limit of the range found in Spain [23], especially as they were among the eight provenances identified by the producers, either in cultivated or wild plants. These results show the importance of producers support in identifying plants with high-sugar fruit, thereby enhancing fermentation yield to produce brandy or liqueur [14,68].

Another potential confounding factor for differences that can be found in fruit production and sugar content is related to years of high vs. low fruit production (harvest vs. counter-harvest years). Ruiz-Rodríguez et al. [23] found that the sugar content, mainly fructose and glucose, was dependent on the year of harvesting (three consecutive years studied). Similar results were reported by Molina et al. [69], who studied the fruit production of *A. unedo*, over two consecutive years. However, Seker & Toplu [28] found no significant differences in fructose, glucose and sucrose in five natural areas for two consecutive years. To avoid this confounding factor, in our study fruits were collected in the same year and in autumn (mature stage fruits). If the stage of the fruit influences the sugar content, as shown by Alarcão-e-Silva et al. [29], it can also be a potential confounding factor. These authors found that fructose content ranged from 2.33% to 20.8% (*w*/*w* d.w.) in unripe and ripe fruits, respectively, while glucose ranged from 3.95% to 12.5%, but no significant differences in sucrose content (8.77–8.68%).

In our study, the southern provenances integrated into the *Accentuated Mesomediterranean* (xerothermic index-based classification) [48], showed the highest mean values, particularly in the ODM-Cortes Pereira provenance (fructose 17.01 ± 1.46%, reducing sugars of 26.56 ± 2.31% and total sugar of 27.07 ± 2.30% *w*/*w* f.w. In this climatic region are included ten provenances (71.4%, 133 accessions, and in eight of these ten provenances (57.1%, 84 accessions), the accessions, both cultivated and wild plants, were identified with the owners’ support. The provenances included in this climatic region are in the center and southern regions of Portugal, except for two (*Barrocal* and *S. Mamede*). *Barrocal* (the southernmost provenance) shows a high xerothermic index (number of dry days; 118.18) and belongs to the *Attenuated Thermomediterranean* region. On the other hand, *S. Mamede* is within *Attenuated Mesomediterranean* region, due to the oceanic influence, associated with altitude (mountain located inland of Portugal but preceded by a flat extension, Podzois).

Our study shows the lowest reducing sugar values in the northernmost provenances (*TM-FE, Fisgas de Ermelo* and *TM-Mosteiró*) and in the southernmost provenance (*Barrocal*). The lowest values in the northernmost provenances are associated with reduced photosynthesis, which is a consequence of the diminished photoperiod at higher latitudes and the lower temperatures experienced during fruit maturation period [41]. Doukani & Tabak [31] determined the total and reducing sugars from two different origins (Tiaret and Tlemcen/Atlas Mountain) in Algeria, using a colorimetric method. The observed values were 8.9–14.0% and 7.0–9.3% (*w*/*w* f.w.), respectively, quite inferior to our mean values of 18.5% and 17.8% (*w*/*w* f.w.), for total and reducing sugars, respectively, probably due to the photosynthesis reduction linked to the altitude and temperature, as we observed in the northern provenances. Similar results are also reported by Boussalah et al. [25] on the Atlas Mountains, in Algeria (4 areas of high altitude), where fructose and glucose values range from 5.5%–8.4% and 2.9–5.2% (*w*/*w* f.w.), respectively. In contrast, the lower sugar contents in *Barrocal* are associated with the highest number of dry days/xerothermic index, indicating low water availability for photosynthesis during growth active period (spring and summer) [48,62]. In addition, the region is characterized by calcareous soils, rich in clay, which tend to retain water, reducing its bioavailability. Different authors report that sugar content and fruit production dependence on environmental conditions, particularly in what concerns water availability and summer drought [23,69].

According to the xerothermic index-based classification) [48], the lowest fructose values were observed in the *Attenuated Thermomediterranean*, the southernmost provenance, *Barrocal* (7.89% *w*/*w* f.w.; with high number of dry days, 118.18), and no significant differences were observed compared to the *Sub-Mediterranean* climatic region, of the northmost provenance, *TM-FE* (8.66%). This provenance shows the second lowest fructose value despite the number of the lowest dry days (42.40), because of the highest annual precipitation value (1390.5 mm, compared to *Barrocal*, 697.0 mm), lower temperature and photoperiod during autumn affecting flowering, and particularly, the fruit maturation [41,59]. Similar results were obtained by Molina et al. [69], who reported differences in fruit production and the number of fruits per branch over the years due to the risk of frost and low minimum temperatures in autumn. In this season, the flowering phase (autumn-0), the subsequent production and fruit maturation (autumn-0 + 1), as well as the ripening of the previous year’s fruits are involved and negatively affected, which is consistent with our results.

On the other hand, according to the climatic classification of Thornthwaite [49], the highest mean values of reducing and total sugars were observed in the 15 accessions of *ODM-Cortes Pereira* provenance classified as C1B′2s2a′, exhibiting significantly higher levels compared to all other provenances. However, no significant differences were observed in the fructose content when compared to the provenances *ODM-Cerca dos Pomares* and *Cadaval*, both classified as C2B′2sa′, according to the Thornthwaite climatic classification [49]. Conversely, according to the Thornthwaite method [49], the lower results for fructose, reducing and total sugars were observed in regions B4B′2sa′ and B3B′2s2a2, corresponding to the northernmost provenances of this study, namely *TM-FE* and *TM-Mosteiró*. Both provenances show a humid climate with a high-water index (B4, B3), with moderate (s) to large (s2) water deficit in summer, respectively *TM-FE* and *TM-Mosteiró*. These results confirm those previously observed and are in line with the conclusions of the xerothermic index [48]. However, unlike the xerothermic index climate classification [48], Thornthwaite’s climate classification [49] does not discriminate the lower results observed in *Barrocal* with the highest number of biologically dry days [48,62], since it was assembled in the same region C2B′2s2a′ as the *ODM-NR* and *ODM-Pomba* provenances. It therefore appears that the xerothermic index establishes a better relationship between climate classification and sugar content (in 14 provenances, N = 204). In addition, the xerothermic index is considered in the literature [48] to be the most accurate for the Mediterranean climates, as it distinguishes regions according to the number of biologically dry days, which could explain the results of lower fructose content in *Barrocal*, despite its southern location. This correlation found allows discriminate potential of the diverse provenances in terms of fruit sugar content, thereby providing an effective instrument for the main regional constraints in the pursuit of optimal solutions for the establishment of orchard or the management of natural areas with wild plants for fruit production. Therefore, for species intolerant to excess water, such as *A. unedo* [70], the results of this study suggest an additional care for the local selection for the orchards, particularly in limestone regions or areas with soils showing higher content of clay. In the northernmost sites, to mitigate the high precipitation, low temperature and reduced photoperiod, orchards or wild plants should be oriented S-SW, in areas not subject to flooding, with wind protection, and not under the canopy to promote entomophilous pollination in the 1st autumn, active fruit growth during the spring-summer seasons, and finally fruit ripening in the 2nd autumn [26,41].

## 5. Conclusions

To survey and collect the genetic diversity of cultivated and wild plants of *A. unedo* for subsequent characterization of their sugar content, a total of 204 accessions were prospected and fruits were harvested in 14 sites/provenances in Portugal.

The predominant sugar in fruit was fructose. The presence of sucrose was residual, inducing a content of total sugars equivalent to that of reducing sugars. Fructose content in fruits is relevant for its commercialization as transformed products (brandy and others) and for consumption as a fresh fruit.

The lowest recorded values of sugar were observed in three provenances: the two northernmost locations (*TM-FE* and *TM-Mosteiró*) and the third, the *Barrocal* (the southernmost provenance and situated in a limestone zone). In the northern regions, the low sugar contents are attributable to elevated precipitation, low temperature, and higher latitude (photoperiod reduction), which, despite higher soil water availability, result in a reduction in sugar synthesis, particularly during the phase of fruit maturation (autumn). In the southern region, the low sugar contents are due to the number of dry days, linked to lower soil water availability, particularly in limestone zones (due to water retention by clay-rich soils). The findings of this study demonstrate the significance of investigating the relationship between sugar content, edaphoclimatic characteristics and climatic classification by xerothermic index. This is with a view to enhancing fruit production, providing guidance on the establishment of new orchards and facilitating the restoration of natural areas. This has the potential to enhance outcomes such as soil erosion protection, wildfire spread prevention and reforestation programs, as a resilient species that supports other autochthonous tree species in the subsequent stages of ecological succession.

The climatic classification by xerothermic index proved an effective tool for distinguishing the potential of each provenance regarding fruit sugar content production, as well as for identifying regional limitations. This approach offers valuable insights into the establishment of new orchards and management of natural areas. So, it is recommended that orchards or natural regions in the northernmost regions be oriented S-SW, in areas not subject to drenching, with wind protection, and not under canopy. In contrast, for the southernmost regions, particularly limestone zones, orchards or natural regions should be in areas with higher water availability, without drenching, and probably under canopy.

For future research, we intend to focus on two relevant points: the full characterization of the accessions and the selection of plant material to continue the strawberry tree breeding program. Work is underway to evaluate the relationship between sugar content and other productivity parameters, such as production (kg/tree), number of fruits per bunch and fruit firmness to facilitate harvesting, fruit size, total soluble solids, sclerenchyma reduction, especially for the fresh fruit market, and finally its relationship with genetic diversity. As far as the plant material is concerned, the breeding program aims to select the best accessions adapted to different ecological conditions and the most genetically diverse for establishment in the seed-production orchard. Last but not least, the remaining accessions will be propagated for establishment in field as an ex situ reserve for the conservation of genetic resources.

## Figures and Tables

**Table 1:** Identification of the number of accessions by provenance and their characterization by sugar content.

Local/Provenance	N	Maltose	Sucrose	Glucose	Fructose	Reducing Sugar	Total Sugar
		(Mean ± SE g/100 g, Fresh Pulp Fruit)					
TM-Fisgas Ermelo	6	0.87 ± 0.30 d	0.00 ± 0.00 c	4.33 ± 0.10 bcd	8.66 ± 0.43 def	13.86 ± 0.71 cde	13.86 ± 0.71 de
TM-Mosteiró	19	1.24 ± 0.23 cd	0.12 ± 0.07 b	3.98 ± 0.11 cd	8.54 ± 0.32 ef	13.77 ± 0.55 de	13.89 ± 0.58 e
São Mamede	26	0.63 ± 0.13 d	0.85 ± 0.11 b	5.13 ± 0.55 bcd	10.42 ± 1.14 cdef	16.18 ± 1.76 de	17.04 ± 1.81 de
Penamacor	24	0.61 ± 0.14 d	0.74 ± 0.12 b	5.14 ± 0.22 bc	11.70 ± 0.39 bcd	17.45 ± 0.58 cd	18.19 ± 0.63 cd
Penha Garcia *	2	0.00 ± 0.00 e	0.00 ± 0.00 c	4.13 ± 0.04 bcd	**12.26 ± 0.20 abcd**	16.39 ± 0.16 bcd	**16.39 ± 0.16 abcd**
Cadaval *	4	0.81 ± 0.27 d	**0.45 ± 0.45 ab**	4.72 ± 0.29 bcd	**12.16 ± 0.61 abcd**	**17.70 ± 1.03 abcd**	**18.15 ± 0.73 abcd**
Monchiqu e *	25	0.67 ± 0.13 d	**1.58 ± 0.15 a**	**6.80 ± 0.26 a**	**12.29 ± 0.61 abcd**	**19.77 ± 0.88 abc**	**21.34 ± 0.97 ac**
ODM-Pomba *	16	2.08 ± 0.16 bc	0.16 ± 0.09 b	4.91 ± 0.28 bcd	**12.66 ± 0.60 abc**	**19.65 ± 0.86 abc**	**19.81 ± 0.89 abcd**
ODM-Cerca Pomares *	3	0.96 ± 0.50 cd	0.32 ± 0.32 b	3.77 ± 0.11 bcd	10.82 ± 0.06 bcd	15.54 ± 0.43 bcd	15.86 ± 0.61 bcd
ODM-Trancão *	9	2.06 ± 0.30 bc	0.32 ± 0.16 b	4.16 ± 0.30 bcd	11.31 ± 0.61 bcd	17.54 ± 0.85 bcd	17.85 ± 0.88 bcd
ODM-Nave Redondo *	10	**3.30 ± 0.96 ab**	**0.66 ± 0.36 ab**	**6.03 ± 0.40 ab**	**15.33 ± 1.28 ab**	**24.66 ± 1.63 ab**	**25.31 ± 1.57 ab**
ODM–Cortes Pereira *	15	**3.73 ± 0.53 a**	0.51 ± 0.17 b	**5.82 ± 0.39 ab**	**17.01 ± 1.46 a**	**26.56 ± 2.31 a**	**27.07 ± 2.30 a**
Caldeirão	25	0.41 ± 0.12 d	**0.97 ± 0.14 ab**	4.93 ± 0.11 b-d	11.78 ± 0.33 b-d	17.12 ± 0.43 cd	18.08 ± 0.45 cd
Barrocal	20	1.02 ± 0.15 d	0.39 ± 0.14 b	4.03 ± 0.25 cd	7.89 ± 0.55 f	12.95 ± 0.79 e	13.34 ± 0.84 e
Total/Global mean	204	1.25 ± 0.10	0.67 ± 0.05	5.07 ± 0.11	11.49 ± 0.28	17.81 ± 0.43	18.47 ± 0.44

* Cultivated and wild plants identified with the Producers’ support. Values are expressed as Mean ± SE% (f.w.); different letters in columns indicate statistically significant differences (*p* < 0.05) according to Tukey multiple range test. Bold values show the highest value and those that are not significantly different.

**Table 2:** Identification of the number of *A. unedo* accessions (N) and edaphoclimatic characterization of the respective provenance in Portugal.

Local/Provenance	N	Lithology ^1^	pH ^2^	T _annual_ (°C) ^3^	P _annual_ (mm) ^3^	Humidity Index	Xeroth. Index	Climatic Classification ^4^	Climatic Classif. ^5^
TM-Fisgas Ermelo/FE	6	Shales, Quarzites	2	13.4	1390.5	111.51	42.40	Sub-Mediterranean	B_4_ B′_2_ s a′
TM-Mosteiró	19	Shales	3	13.4	1128.1	84.69	60.96	Attenuated Mesomed ^4.1^	B_3_ B′_2_ s_2_ a′
São Mamede	26	Shales, Sandst. ^1.1^	2	15.2	852.4	45.41	71.38	Attenuated Mesomed ^4.1^	B_1_ B′_2_ s_2_ a′
Penamacor	24	Shales, Quarzites	2	14.4	838.2	47.80	98.64	Accentuated Mesomed ^4.1^	B_1_ B′_2_ s_2_ a′
Penha Garcia *	2	Shales	3	14.4	838.2	47.80	98.64	Accentuated Mesomed ^4.1^	B_1_ B′_2_ s_2_ a′
Cadaval *	4	Gonglomerates ^1.2^ Sand ^1.1^	3	15.0	777.6	40.20	86.64	Accentuated Mesomed ^4.1^	C_2_ B′_2_ s a′
Monchique *	25	Syenite & Shale	2	15.1	949.0	60.22	87.36	Accentuated Mesomed ^4.1^	B_1_ B′_2_ s_2_ a′
ODM-Pomba *	16	Shale, graywacke	3	15.8	715.7	34.27	87.52	Accentuated Mesomed ^4.1^	C_2_ B′_2_ s_2_ a′
ODM-Cerca Pomares *	3	Shale, graywacke	3	15.0	760.5	35.69	88.96	Accentuated Mesomed ^4.1^	C_2_ B′_2_ s a′
ODM-Trancão *	9	Shale, graywacke	3	15.0	571.9	15.73	85.04	Accentuated Mesomed ^4.1^	C_1_ B′_2_ s a′
ODM-Nave Redondo *	10	Shale, graywacke	3	15.1	748.9	40.03	90.56	Accentuated Mesomed ^4.1^	C_2_ B′_2_ s_2_ a′
ODM-Cortes Pereira *	15	Shale, graywacke	3	15.8	623.1	22.08	83.52	Accentuated Mesomed ^4.1^	C_1_ B′_2_ s_2_ a′
Caldeirão	25	Shale, graywacke	3	15.9	866.7	51.40	95.90	Accentuated Mesomed ^4.1^	B_1_ B′_2_ s_2_ a′
Barrocal	20	Limestone	4	15.9	697.0	33.96	118.18	Attenuated Thermomed ^4.2^	C_2_ B′_2_ s_2_ a′

* Cultivated and wild plants identified with the Producers’ support. ^1^ Lithology according to lithological map [44] associated, in general (according to Soil Classification WRB [45]), to Leptosols and Cambisols [46]. ^1.1^ Shales and sandstones; ^1.2^ conglomerates and sandstones. ^2^ pH soil classification mean per provenance: 1-very low (<4); 2-low (4–5.4); 3-medium (5.5–7.4); 4-high (7.5–9); 5-very high (>9). ^3^ Annual averages of temperature (°C) and precipitation (mm), corresponding to 1951–1980 period [47]. ^4^ Climatic classification according to xerothermic index [48]. ^4.1^ Mesomediterranean; ^4.2^ Thermomediterranean. ^5^ Climatic classification according to Thornthwaite [49] present in the table: 1st letter (Hydric index–IH): (i) humid and rainy climates B4, B3 e C2—respectively very humid, humid and subhumid vs. (ii) dry climates C1–Dry subhumid. 2nd letter (Thermal efficiency (∑ PET): B′2—mesothermic. 3rd letter for mediterranean climate: (i) Humid and subhumid climate group (1st Letter A, B or C2):–s (moderate water deficiency in summer); s2 (severe scarcity of water in the summer); (ii) Dry climate group (1st letter C1 or D): s (moderate excess water in winter); s2 (high water excesses in the winter season). 4th letter (concentration of thermal efficiency in the hot season): a′–small concentration of thermal efficiency during summer.

**Table 3:** Identification of the number of accessions by groups of the climate classification according to xerothermic index [48] and their characterization on sugar content.

Climatic Classification	N	Maltose	Sucrose	Glucose	Fructose	Reducing Sugar	Total Sugar
		(Mean ± SE g/100 g, Fresh Pulp Fruit)					
Accentuated Mesomediterranean	133	**1.42 ± 0.15 a**	0.78 ± 0.07 a	**5.40 ± 0.12 a**	**12.79 ± 0.29 a**	**19.61 ± 0.46 a**	**20.39 ± 0.47 a**
Attenuated Mesomediterranean	45	0.89 ± 0.13 b	0.55 ± 0.09 a	4.64 ± 0.33 ab	9.63 ± 0.68 b	15.16 ± 1.05 b	15.71 ± 1.09 b
Attenuated Thermomediterranean	20	1.02 ± 0.15 ab	0.39 ± 0.14 a	4.03 ± 0.25 c	7.89 ± 0.55 c	12.95 ± 0.79 b	13.34 ± 0.84 b
Sub-Mediterranean	6	0.87 ± 0.30 b	0.00 ± 0.00 a	4.33 ± 0.10 bc	8.66 ± 0.43 bc	13.86 ± 0.71 b	13.86 ± 0.71 b
Total/Global mean	204	1.25 ± 0.10	0.67 ± 0.05	5.07 ± 0.11	11.49 ± 0.28	17.81 ± 0.43	18.47 ± 0.44

Values are expressed as Mean ± SE% (f.w.); different letters in columns indicate statistically significant differences (*p* < 0.05) according to Tukey multiple range test. Bold values show the highest value and those that are not significantly different.

**Table 4:** Identification of the number of accessions by groups of the climate classification according to Thornthwaite [49] and their characterization on sugar content.

Climatic Classification	N	Maltose	Sucrose	Glucose	Fructose	Reducing Sugar	Total Sugar
		Mean ± SE (g/100 g, Fresh Pulp Fruit)					
B4 B′2 s a′	6	0.87 ± 0.30 cde	0.00 ± 0.00 ab	4.33 ± 0.10 ab	8.66 ± 0.43 bc	13.86 ± 0.71 bc	13.86 ± 0.71 bc
B3 B′2 s2 a′	19	1.24 ± 0.23 cd	0.12 ± 0.07 b	3.98 ± 0.11 b	8.54 ± 0.32 c	13.77 ± 0.55 c	13.89 ± 0.58 c
B1 B′2 s2 a′	102	0.57 ± 0.06 e	1.02 ± 0.07 a	5.47 ± 0.18 a	11.55 ± 0.35 b	17.59 ± 0.54 b	18.61 ± 0.57 b
C1 B′2 s a′	9	2.06 ± 0.30 b	0.32 ± 0.16 ab	4.16 ± 0.30 ab	11.31 ± 0.61 bc	17.54 ± 0.85 bc	17.85 ± 0.88 bc
**C1 B′2 s2 a′**	15	3.73 ± 0.53 a	0.51 ± 0.17 ab	5.82 ± 0.39 a	**17.01 ± 1.46 a**	**26.56 ± 2.31 a**	**27.07 ± 2.30 a**
**C2 B′2 s a′**	7	0.87 ± 0.24 de	0.39 ± 0.27 ab	4.31 ± 0.25 ab	**11.59 ± 0.43 abc**	16.77 ± 0.72 bc	17.17 ± 0.65 bc
C2 B′2 s2 a′	46	1.89 ± 0.25 bc	0.37 ± 0.10 ab	4.77 ± 0.20 ab	11.17 ± 0.61 bc	17.83 ± 0.89 bc	18.19 ± 0.91 bc
Total/Global mean	204	1.25 ± 0.10	0.67 ± 0.05	5.07 ± 0.11	11.49 ± 0.28	17.81 ± 0.43	18.47 ± 0.44

Values are expressed as Mean ± SE% (f.w.); different letters in columns indicate statistically significant differences (*p* < 0.05) according to Tukey multiple range test. Bold values show the highest value and those that are not significantly different. Following several iterations of the linear regression analysis, the results indicate a stronger correlation between the total sugar content of fruits and the climate classification according to the xerothermic index than that indicated by the Thornthwaite method. In conclusion, fourteen variables, related to edaphoclimatic characteristics such as: local/provenance, annual averages of precipitation and temperature, altitude, xerothermic index, sun exposure, light conditions, soil texture, soil pH, longitude & latitude, allocation (inland vs. coast), humidity index and climatic classification (according to xerothermic index) were chosen. Table 5 shows the results of the multiple regression analysis of the dependent variable, total sugar content (Log_10_% f.w.), as a function of the above referred fourteen variables, showing a correlation coefficient (R) of 0.671 for the total of 204 sample accessions.

**Table 5:** Multiple linear regression analysis for total sugar content, assessed by the dependent variable Total Sugar% f.w. (Log_10_) as a function of the different independent variables ^1^. Regression Summary for dependent variable: Log_10_ Total Sugar%, R = 0.67101272, R^2^ = 0.45025808, Adjusted R^2^ = 0.40953645, *F* (14,189) = 11.057, *p* < 0.00000, Std. Error of estimate: 0.10452.

N = 204	B *	Std. Err. of b *	b	Std. Err. of b	t (189)	*p*-Value
Intercept			8.936646	8.271868	1.08037	0.281356
Local/Prov. *	**0.36478**	**0.178853**	**0.014216**	**0.006970**	**2.03954**	**0.042788**
P annual (mm) *	**−4.17974**	**1.371076**	**−0.003352**	**0.001100**	**−3.04851**	**0.002629**
T annual (°C)	0.20738	0.116945	0.029438	0.016600	1.77335	0.077782
Altitude (m)	−0.23436	0.149311	−0.000202	0.000129	−1.56964	0.118171
Xerothermic index *	**−0.40376**	**0.196952**	**−0.003293**	**0.001606**	**−2.05014**	**0.041732**
Sun Exp. (1–8)	−0.03605	0.066087	−0.002118	0.003883	−0.54542	0.586106
Light (1–3)	0.00809	0.092425	0.002102	0.024016	0.08752	0.930354
Soil Texture (1–11)	0.18024	0.094916	0.009293	0.004894	1.89900	0.059088
pH (1–5) *	**−0.46739**	**0.195788**	**−0.093108**	**0.039002**	**−2.38724**	**0.017962**
Longitude	−0.05647	0.287082	−0.013405	0.068146	−0.19671	0.844268
Latitude	0.33393	0.212757	0.030505	0.019436	1.56956	0.118191
Humid Index (Ihu) *	**3.63697**	**1.304984**	**0.024660**	**0.008848**	**2.78698**	**0.005863**
Climat. Clas. Xero. * Ind. *	**−0.22129**	**0.096013**	**−0.031799**	**0.013797**	**−2.30479**	**0.022265**
Inland vs. Coast	−0.17945	0.273031	−0.054265	0.082564	−0.65724	0.511823

^1^ Independent variables: local/provenance *, annual averages of precipitation * and temperature, altitude, xerothermic index *, sun exposure, light conditions, soil texture, soil pH *, longitude & latitude, allocation (inland vs. coast), humidity index *, climatic classification (according to xerothermic index) *. Bold values show independent variables * that contribute significantly (*p* < 0.05) to the total sugar content (dependent variable).

## Data Availability

Data are contained within the article.
